# Effects of Reef Proximity on the Structure of Fish Assemblages of Unconsolidated Substrata

**DOI:** 10.1371/journal.pone.0049437

**Published:** 2012-11-16

**Authors:** Arthur L. Schultz, Hamish A. Malcolm, Daniel J. Bucher, Stephen D. A. Smith

**Affiliations:** 1 National Marine Science Centre, Southern Cross University, Charlesworth Bay, Coffs Harbour, New South Wales, Australia; 2 Aquatic Ecosystem Research, NSW Department of Primary Industries, Coffs Harbour, New South Wales, Australia; 3 Marine Ecology Research Centre, School of Environment, Science and Engineering, Southern Cross University, Lismore, New South Wales, Australia; National Institute of Water & Atmospheric Research, New Zealand

## Abstract

Fish assemblages of unconsolidated sedimentary habitats on continental shelves are poorly described when compared to those of hard substrata. This lack of data restricts the objective management of these extensive benthic habitats. In the context of protecting representative areas of all community types, one important question is the nature of the transition from reefal to sedimentary fish assemblages. We addressed this question using Baited Remote Underwater Videos (BRUVs) to assess fish assemblages of sedimentary habitats at six distances from rocky reefs (0, 25, 50, 100, 200, and 400 m) at four sites in subtropical eastern Australia. Distance from reef was important in determining fish assemblage structure, and there was no overlap between reef sites and sedimentary sites 400 m from reef. While there was a gradient in assemblage structure at intermediate distances, this was not consistent across sites. All sites, however, supported a mixed ‘halo’ assemblage comprising both reef and sediment species at sampling stations close to reef. BRUVs used in conjunction with high-resolution bathymetric and backscatter spatial data can resolve differences in assemblage structure at small spatial scales (10s to 100s of metres), and has further application in unconsolidated habitats. Unless a ‘reef halo’ assemblage is being examined, a minimum of 200 m but preferably 400 m distance from any hard substrate is recommended when designing broader-scale assessments of fish assemblages of sedimentary habitats.

## Introduction

Despite the fact that sedimentary environments are the most extensive subtidal habitat of the world’s oceans [1], research on fish assemblages in shallow coastal waters has primarily been conducted on reefal habitats, and understanding of fish assemblages of unconsolidated habitats is relatively poor [2]–[3]. Sedimentary habitats have often been characterised as less topographically complex and more physically stressed than reef environments [4], providing fewer niches and lower primary production than reef habitats [5]. However, many species utilise this habitat, and species richness may be comparable to that found on adjacent reefs [6].

The lack of available data on unconsolidated habitats and associated biotic patterns restricts the objective management of these habitat types. Marine Protected Areas have been developed worldwide with conservation of biodiversity as a primary objective [7], and a representative sample of all habitats and biota is required if conservation outcomes are to be met [8]–[9]. The use of habitat as a surrogate for biodiversity is often unavoidable, as detailed species and assemblage inventories are rare [10]–[12]. However, this will only be effective if biotic patterns consistently and predictably match physical attributes, which requires testing with biological surveys [13].

In New South Wales (NSW) marine parks, a Habitat Classification Scheme (HCS) is employed as a surrogate for biodiversity, and uses depth and habitat type (such as ‘rocky reef’ and ‘unconsolidated sediment’) as primary categories. While the HCS has been tested for effectiveness against patterns of fish assemblage structure on rocky reefs [14]–[16] this has not been assessed for fish assemblages of unconsolidated habitats.

Many species found on unconsolidated habitats are morphologically and behaviourally adapted to this environment, posing problems for visual surveys. For example, fish such as those from the family Platycephalidae bury themselves in the sediment to ambush prey [17], and sillaginids are highly mobile, and very sensitive to sound and vibration [18]. These traits, combined with the practical depth limitations for SCUBA-based visual census (see [19] for a review), creates logistical difficulties in accurately and non-destructively sampling entire fish assemblages in these environments. For this reason, destructive trawl sampling has often been employed [20]–[21], but this method is not compatible with marine park objectives.

Baited Remote Underwater Video (BRUV) has been developed to reduce constraints involved in fish assemblage research by SCUBA methods [22]–[23]. BRUVs have been successfully used in many hard substrate environments to assess fish assemblages [24]–[25] but have only recently been applied to shallow unconsolidated habitats [26]–[29]. The effectiveness of BRUVs for assessing abundance of carnivorous fish otherwise shy of divers is well documented [22], [30], and [26] suggest that BRUVs may have a particular role in studies of larger elasmobranchs or teleost species of special conservation interest that other sampling methods do not reliably record. There is also great application for BRUVs in habitats below depths accessible by SCUBA [31], and they have been widely employed in deep-sea research since the 1970s [32]–[33].

Not only are data lacking for fish assemblages of unconsolidated habitats in general, but also relatively little is known about linkages between unconsolidated and reefal habitats (but see [34]). ‘Top down’ influences by consumers foraging across multiple habitats can be strong. Predators [34] and herbivores [35] will often most intensively forage in areas closest to their primary shelter habitat. Predatory species associated with reef habitats may effectively forage across adjacent sedimentary environments, while cryptic species and ambush predators may concentrate predation effort close to hard substrata (e.g. [36]).

Gradients in assemblage structure have been inferred by studies demonstrating a reduction in predators [34], [37]–[38], and an increase in prey densities [39]–[42] with increasing distance from reef. Decreases in the abundance and richness of herbivorous reef fish with increasing distance from reef have also been documented, with associated decreases in consumption rates of detached macroalgae [43] and seagrass [35], [44]. Each of these studies demonstrated a halo of sedimentary habitat surrounding a reef in which parameters change in response to distance from reef. However, all of these studies focus on a subset of the reef fish assemblage, or on single species and associated food densities, and did not attempt to describe patterns in the entire assemblage.

The focus of this research was, therefore, to investigate how reef proximity influences fish assemblages of unconsolidated substrata. We deployed replicated BRUVs in two sites at each of two locations and a fixed depth range (20–30 m) to: 1) quantify changes in fish assemblage structure with increasing distance from reef, and 2) identify the species that drive differences in assemblage structure across this gradient. We also tested the spatial consistency of these patterns between and within two locations at the scale of 100s of metres to kilometres.

## Materials and Methods

### Study Area and Sampling Design

The Solitary Islands Marine Park (SIMP) is situated on the north coast of New South Wales, in the Tweed-Moreton Bioregion, and covers an area of approximately 71,000 hectares. The region lies within a broad ecotone between tropical and temperate assemblages, supporting high diversity with consequently high conservation status (e.g. [14], [45]–[46]). The SIMP has extensive, though patchy, reef habitats, but the majority (∼80%) of benthic habitat is unconsolidated [47]–[48].

**Figure 1:**
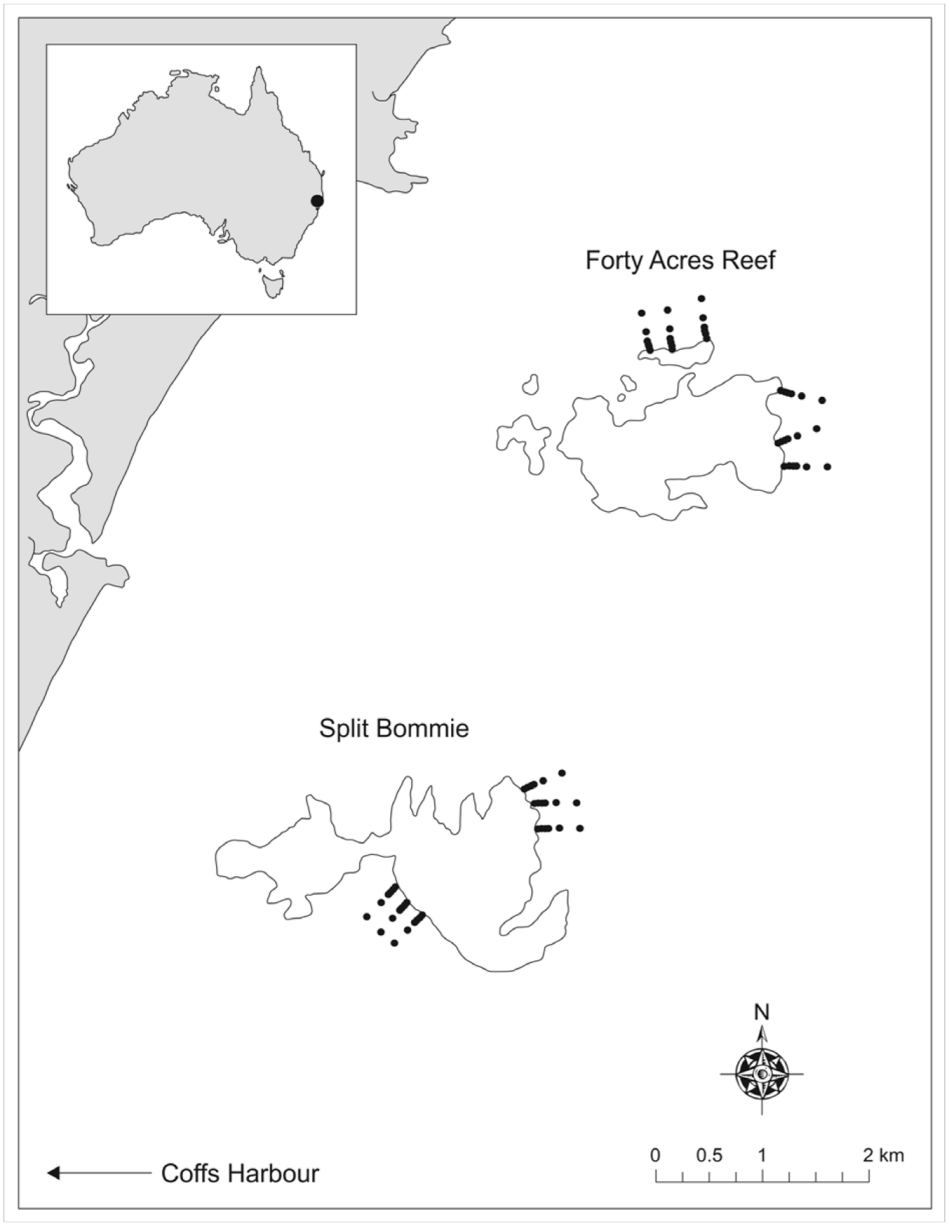
The location of the 72 individual BRUV deployments at Forty Acres Reef and Split Bommie Reef in the Solitary Islands Marine Park (SIMP), New South Wales, Australia.

A three-way, partially nested design was used to assess demersal fish assemblages at two locations within the SIMP using BRUVs. Sites were selected using existing high-resolution bathymetric and backscatter spatial data [47] (Fig. 1), using ArcMap, to ensure accuracy in establishing distances from reef. Two locations were selected - Forty Acres Reef and Split Bommie - and two sites at each reef were sampled within each location (Fig. 1). Transects were selected at the edge of the reef (0 m) and along a gradient from the reef at 25, 50, 100, 200 and 400 m. Three replicate BRUVs were deployed at each distance from reef for each site within 5–10 minutes of each other. A minimum distance of 200 m was maintained between replicates to ensure independence [25]. Coordinates were uploaded to a handheld GPS which was then used in the field to position BRUVs. Soak time was 30 min, and only samples with a full field-of-view were retained for analysis. Sampling was conducted between March-May 2011 (Austral autumn).

Each BRUV unit consisted of a Mini-DV video camera with a wide angle lens, in a high-density polyethylene underwater housing with a flat acrylic end lens, an attachment frame, and rope and float system linking the unit to the surface. Approximately 1 kg of pilchard (*Sardinops neopilchardus*) was used to attract fish to the field of view (as per [28]). The bait was mashed into a mesh bag attached to the end of a bait pole, 1.5 m from the front of the camera.

### Analysis

Mini DV video tapes were converted to a digital format (avi) for analysis using Adobe Premiere Elements (Version 10, Adobe Systems Pty Ltd), at a suitable resolution for fish identification (720×576). Files were then analysed using the program Eventmeasure (SeaGIS Pty Ltd, Version 3.31). The identity of each fish species and an index of its relative abundance (MaxN) were recorded. MaxN is the maximum number of individuals of each species within the field of view at any one instant during the 30-min recording. This removed the possibility of double counts of individual fish. As counts reflected relative abundance and not density, we expected data to be robust to variability in estimating this field of view, and all video analysis was performed by the same observer (AS) [25]. Data on relative abundance of all fish species recorded in the study area were compiled to compare assemblage structure with varying distance from reef.

A range of multivariate and univariate analyses were applied using procedures in the PRIMER 6.0 software package [49], including the PERMANOVA+add on [50]. Non-metric multidimensional scaling (nMDS) ordination based on Bray-Curtis similarities of square-root transformed data, was used to visually depict changes in assemblage structure with increasing distance from reef. Permutational multivariate analysis-of-variance (PERMANOVA, [51]) was then used to test the null hypothesis of no difference in assemblage structure with distance from reef, between locations, and between sites nested within locations. A three-way, partially nested design was used, where the factor ‘Distance’ was analysed as a fixed factor with six levels (0, 25, 50, 100, 200 and 400 m), the factor ‘Location’ was a fixed factor with two levels (Split Bommie and Forty Acres), and the factor ‘Site’ was a random factor with two levels nested within ‘Location’. Multivariate dispersion (MVDISP) indices were then calculated [52], with ‘Distance’ as the factor, to generate numerical indices of similarity among replicates at each distance from reef. In these analyses, a greater value indicates a greater dissimilarity between replicates, and a value of zero indicates no difference between replicates.

Where PERMANOVA revealed a statistically significant result, SIMPER analysis was used to determine the species most responsible for differences in assemblage structure [53]. Univariate PERMANOVAs (using Euclidean distance as the similarity measure) were performed for each species identified as contributing strongly to differences across the factors.

## Results

### Fish Assemblage

A total of 76 species from 37 families was observed. Eight species of chondrichthyans were recorded, four sharks and four rays. Osteichthyans were from various trophic groups including planktivores, herbivores and predators. The families Labridae and Platycephalidae were the most speciose (six species each). A total of 1640 individuals were recorded by combining all MaxN data. The most abundant species were the predator *Pagrus auratus* (Sparidae, 364 individuals), the schooling planktivore *Atypichthys strigatus* (Scorpididae, 219 individuals) and the piscivore *Platycephalus longispinis* (Platycephalidae, 180 individuals). Twenty-four species were recorded only once, which is consistent with studies of reef fish in the SIMP [15] and elsewhere [54] which found many species are rarely recorded.

### Multivariate Analyses

**Figure 2:**
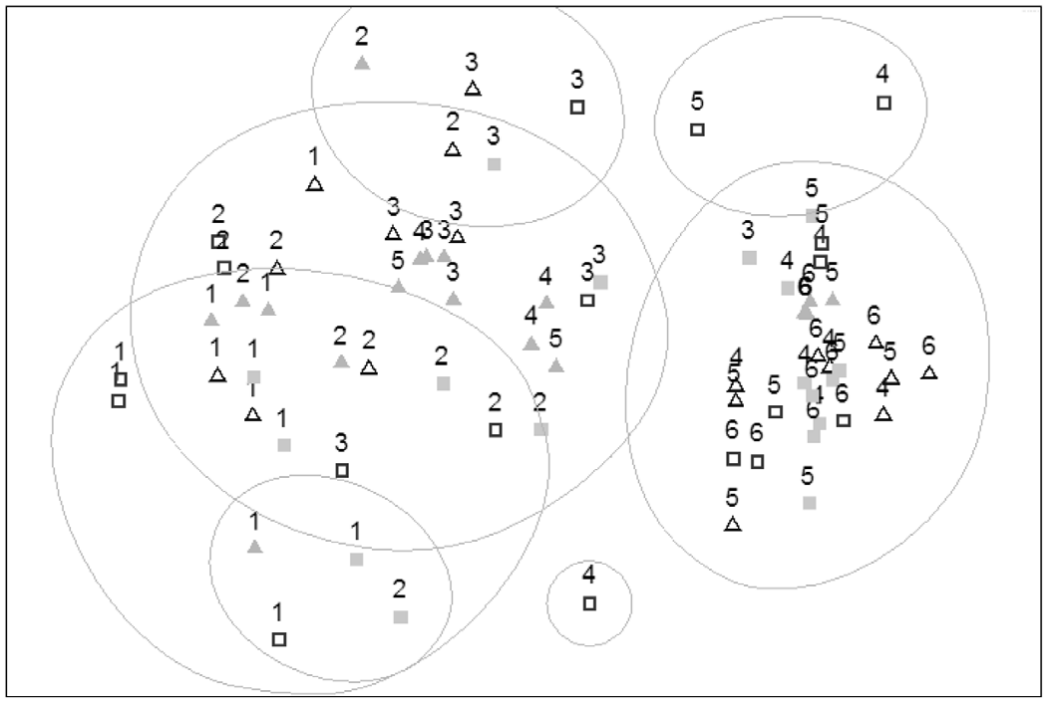
Non-metric multi-dimensional scaling (nMDS) ordination showing the relationship among fish assemblages from four sites and six distances from reef. Data were square-root transformed prior to analysis. Lines represent 30% similarity. Stress = 0.14. Grey triangles = Forty Acres East, clear triangles = Forty Acres North, grey squares = Split Bommie North, clear squares = Split Bommie West. 1 = 0 metres, 2 = 25 metres, 3 = 50 metres, 4 = 100 metres, 5 = 200 metres, 6 = 400 metres.

There was an obvious gradient in community structure with increasing distance from reef (Fig. 2). However, only the 400 m sites showed a tight grouping (with>30% similarity), and assemblage structure overlapped across intermediate distances. The greatest separation generally occurred between the 50 and 100 m distances, with most samples from 0, 25 and 50 m grouping to the left, and those from 100–400 m grouping to the right of the plot (Fig. 2). Samples from 100 and 200 m at Forty Acres East were the exception, and grouped to the left of the plot. The two outliers (top right of the plot) recorded only one and two species, respectively. The lone outlier at the bottom of the plot recorded three species, one otherwise not recorded in the study. The results of Multivariate Dispersion (MVDISP) analyses indicated a much stronger similarity between all replicates from 400 m relative to samples from all other distances, which showed similar dispersion (Table 1).

**Table 1:** Index of Multivariate Dispersion (MVDISP) across replicates pooled within each of the six distances from reef.

distance from reef (m)	RD
400	0.407
50	1.054
200	1.082
0	1.087
100	1.151
25	1.220

PERMANOVA revealed significant effects for Distance and Site (Location) (Table 2). However, the highest-order interaction was also highly significant, making it impossible to interpret distance effects without accounting for variation at the Site level. For this reason, separate one-way analyses were performed for each of the four sites and *post-hoc* pairwise contrasts were performed where the effect of distance was significant (Table 3).

**Table 2:** Summary of PERMANOVA results for the analysis of differences in assemblage structure across the different factors.

Source of variation	df	MS	Pseudo-F	*P*
Distance	5	17712	6.3554	**0.001**
Location	1	7684.2	1.3098	0.329
Site (Location)	2	5866.5	3.8861	**0.001**
Distance × Location	5	2817.4	1.011	0.471
Distance × Site (Location)	10	2786.9	1.8461	**0.001**
Residual	48	1509.6		

Significant results are shown in bold.

**Table 3:** Results of *post-hoc* pairwise contrasts of assemblage structure (PERMANOVA) for each pair of distances at each of the four sites.

Contrast	*P* (Perm)			
	FAE	FAN	SBN	SBW
0m vs 25m	0.513	0.304	0.406	0.121
0m vs 50m	**0.001**	**0.001**	**0.001**	**0.001**
0m vs 100m	0.101	**0.001**	**0.001**	**0.001**
0m vs 200m	0.113	**0.001**	**0.001**	**0.001**
0m vs 400m	**0.001**	**0.001**	**0.001**	**0.001**
25m vs 50m	0.307	0.426	0.204	0.805
25m vs 100m	0.301	**0.001**	**0.001**	**0.001**
25m vs 200m	0.204	**0.001**	**0.001**	**0.001**
25m vs 400m	**0.001**	**0.001**	**0.001**	**0.001**
50m vs 100m	0.294	**0.001**	**0.001**	0.276
50m vs 200m	0.324	**0.001**	**0.001**	**0.001**
50m vs 400m	0.001	**0.001**	**0.001**	**0.001**
100m vs 200m	0.676	0.591	0.084	0.801
100m vs 400m	**0.001**	0.178	**0.001**	0.091
200m vs 400m	**0.001**	0.194	**0.001**	**0.001**

Significant values are shown in bold. FAE = Forty Acres East, FAN = Forty Acres North, SBN = Split Bommie North, SBW = Split Bommie West.

Having established that differences in assemblage structure were consistently found across distances at each site, SIMPER analysis was then used to determine which species were primarily responsible for these differences. Ten species were found to be important discriminators (accounting for the first 50% of assemblage differences) across all analyses. Of these, four species, *P. auratus, A. strigatus, P. longispinis* and *Sillago ciliata,* appeared more than three times in pairwise comparisons between distances, so abundances of these were explored further.

### Univariate Analyses

#### Species richness

**Figure 3:**
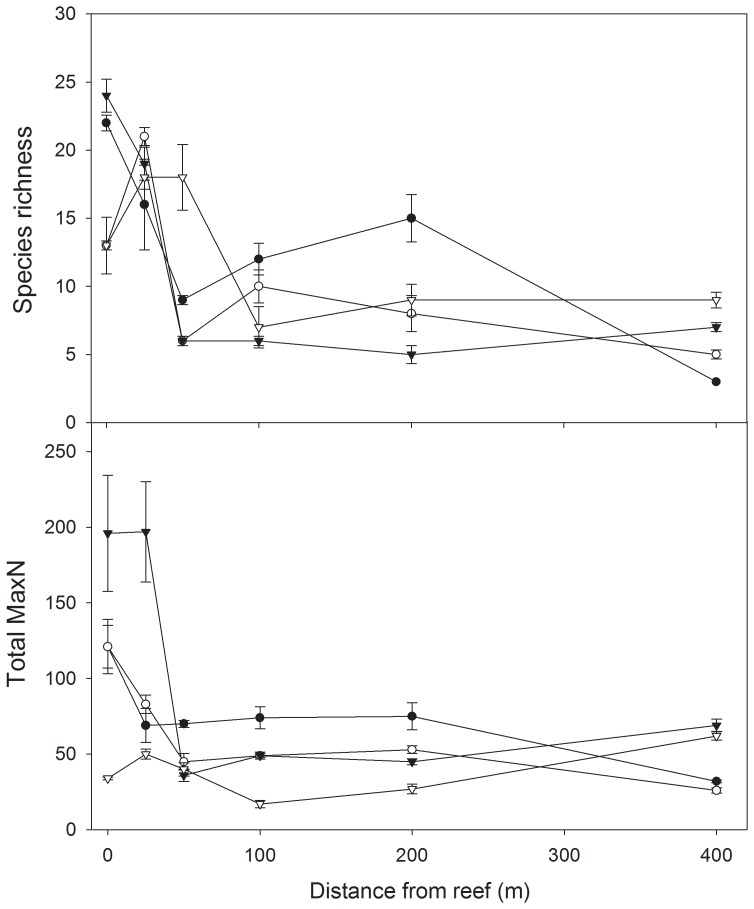
Mean species richness and MaxN (±SE) at four sites and six distances from reef. Black circles = Forty Acres East, clear circles = Forty Acres North, black inverted triangles = Split Bommie North, clear inverted triangles = Split Bommie West.

Trends in mean species richness were similar across three of the four sites, with the highest values at 0 or 25 m, a rapid decline to a distance of 100m, and similar values thereafter (Fig. 3). Forty Acres East displayed a slightly different pattern with relatively high species richness at intermediate distances. PERMANOVA indicated a significant effect for Distance (Table 4, P = 0.012), but not for any of the other terms in the analysis (Table 4). Further analysis using *post-hoc* pairwise tests indicated significant differences between 0 vs 100 m (P = 0.001), 0 vs 200 m (P = 0.001), and 25 vs 400 m (P = 0.012).

**Table 4:** Summary of results (*P*-values) of univariate PERMANOVA for mean species richness and total MaxN, and for the four species determined by SIMPER as being the primary drivers of assemblage differences across the study.

Source	S	Total MaxN	*P. auratus*	*S. ciliata*	*P. longispinis*	*A. strigatus*
Distance	**0.012**	0.106	**0.041**	0.053	**0.005**	0.058
Location	1.000	1.000	0.345	1.000	1.000	1.000
Site (Location)	0.352	**0.004**	0.081	**0.001**	**0.014**	0.23
Distance × Location	0.175	0.111	0.617	0.176	0.465	0.686
Distance × Site (Location)	0.085	0.254	**0.007**	**0.001**	0.106	0.821

Significant results are shown in bold. S = Species richness.

#### Total MaxN

**Figure 4:**
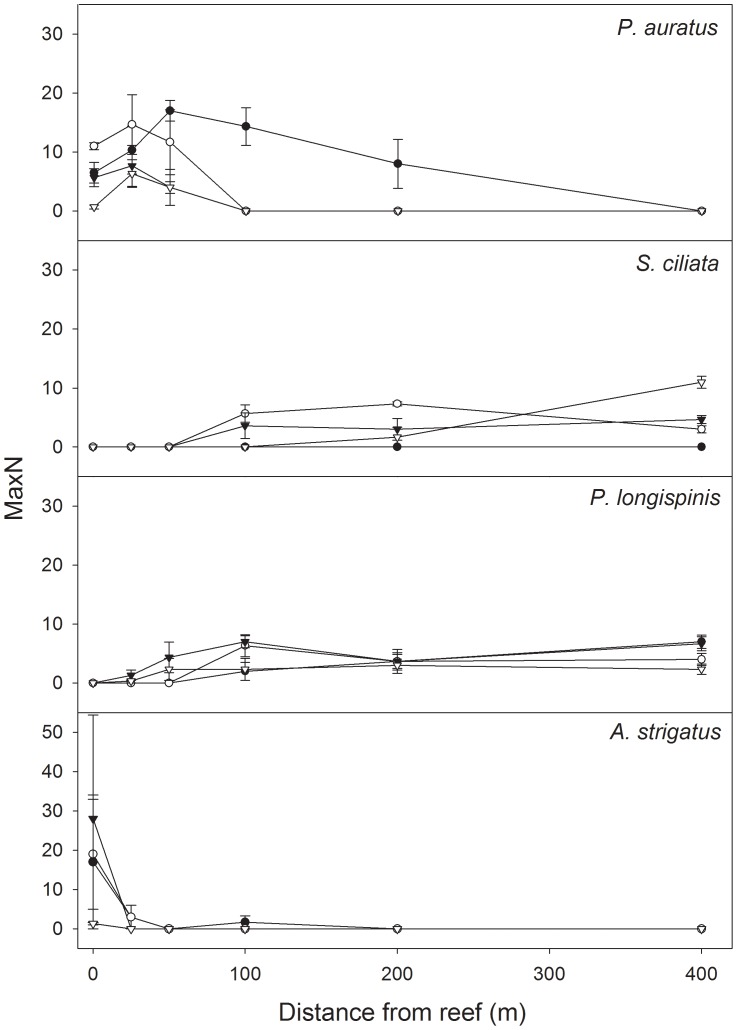
The relative abundance (MaxN) of species most responsible for discriminating between fish assemblages. Mean MaxN (±SE) for the four species determined by SIMPER as being the most important contributors to differences in assemblage structure across distances from reef. (Note the difference in scale for *A. strigatus*) Black circles = Forty Acres East, clear circles = Forty Acres North, black inverted triangles = Split Bommie North, clear inverted triangles = Split Bommie West.

Assessments of differences of total MaxN were complicated by very patchy distribution of some of the abundant taxa. Thus, at 0 and 25 m for Split Bommie North, very high abundance of the schooling planktivore *A. strigatus* dominated the combined abundance (Figs. 3 and 4). Once the abundance of this species was accounted for, there were no obvious Distance-related effects. Indeed, the only significant term in the PERMANOVA was associated with differences between Sites nested within Location (Table 4, P = 0.004), and, once again, mostly likely due to high abundances of a single species.

#### *P. auratus*

There were few consistent Distance-related trends for *P. auratus*. While there was a tendency for all sites to support greater abundances on, and close to, reefs, moderate abundances were also evident at intermediate distances at Forty Acres East (Fig. 4). *P. auratus* were absent from all sites at 400 m. Because of the significant higher-order interaction in the PERMANOVA (Table 4, Distance × Site (Location), P = 0.007) separate one-way PERMANOVAs were performed for each of the four sites. These revealed significant differences at Forty Acres East (P = 0.01) Forty Acres North (P = 0.011) and Split Bommie West (P = 0.011). However, *post-hoc* pairwise contrasts did not identify consistent trends (Table 5).

**Table 5:** Results of *post-hoc* pairwise contrasts of MaxN data for *P. auratus* and *S. ciliata* for sites where Distance was found to be significant.

Contrast	P (perm)				
	*P. auratus*			*S. ciliata*	
	FAE	FAN	SBW	FAN	SBW
0m vs 25m	0.294	0.602	**0.001**	NA	NA
0m vs 50m	**0.001**	0.695	**0.001**	NA	NA
0m vs 100m	**0.001**	**0.001**	**0.001**	**0.001**	NA
0m vs 200m	0.513	**0.001**	**0.001**	**0.001**	0.319
0m vs 400m	**0.001**	**0.001**	**0.001**	**0.001**	**0.001**
25m vs 50m	0.214	0.572	0.293	NA	NA
25m vs 100m	0.522	**0.001**	**0.001**	**0.001**	NA
25m vs 200m	0.602	**0.001**	**0.001**	**0.001**	0.303
25m vs 400m	**0.001**	**0.001**	**0.001**	**0.001**	**0.001**
50m vs 100m	0.371	**0.001**	**0.001**	**0.001**	NA
50m vs 200m	0.114	**0.001**	**0.001**	**0.001**	0.323
50m vs 400m	**0.001**	**0.001**	**0.001**	**0.001**	**0.001**
100m vs 200m	**0.2**	NA	NA	0.458	0.307
100m vs 400m	**0.001**	NA	NA	0.185	**0.001**
200m vs 400m	**0.001**	NA	NA	**0.001**	**0.001**

Significant values are shown in bold. FAE = Forty Acres East, FAN = Forty Acres North, SBW = Split Bommie West. NA indicates a zero abundance for one or both of the pairs.

#### *S. ciliata*

While some Distance-related trends were evident for *S. ciliata,* these trends were not consistent across all sites. Forty Acres North and Split Bommie North supported moderate abundances at 100, 200 and 400m while, at Split Bommie West, *S. ciliata* was absent at 100 m and in low abundance at 200 m, but recorded the highest abundance of any site at 400 m (Fig. 4). *S. ciliata* was not recorded at Forty Acres East at all, and was absent from all 0, 25 and 50m sites. Again, due to the significant highest-order interaction (Table 4, Distance × Site (Location), P = 0.001), separate one-way PERMANOVAs were performed for the three sites at which *S. ciliata* was recorded, revealing significant differences at Forty Acres North (P = 0.001) and Split Bommie West (P = 0.016). *Post-hoc* pairwise contrasts were also performed for these two sites, but no consistent trends were evident (Table 5).

#### *P. longispinis*

Overall, there was a trend of greater abundance further from reef for *P. longispinis*. There was greater abundance of *P. longispinis* at 100, 200 and 400 m than closer to reef across all sites, with the exception of Split Bommie North, where the 50m site recorded higher abundance than at 200 m (Fig. 4). Low abundance was recorded at 50 m for Split Bommie West, and at 25 m for both Split Bommie sites, but *P. longispinis* was absent from Forty Acres sites at these distances. *P. longispinis* was absent from all reef sites (Fig. 4). PERMANOVA revealed a significant Distance-related effect (P = 0.005), as well as a significant effect for Site nested within Location (P = 0.014), but not for other factors (Table 3).

#### *A. strigatus*

No trends were evident for *A. strigatus* (Fig. 4). This schooling planktivore was abundant at three of the four sites at 0 m, but was patchily distributed between replicates, as demonstrated by large standard errors (Fig. 4). *A. strigatus* was uncommon at 25 m and almost absent from more distant sites (Fig. 4). Despite the very strong decrease in abundance with increasing distance from reef, this was associated with high variability and none of the terms were found to be significant in PERMANOVA (Table 3).

## Discussion

A change in fish assemblage from a ‘rocky reef’ to a ‘sedimentary’ assemblage was demonstrated at all sites in this study, with the greatest separation between 0 and 400 m. At distances<50 m from reef, the assemblage had a strong association with reef. From 100 to 200 m there was a ‘halo’ assemblage that still showed reef influence. The assemblage at 400 m from reef was independent of the adjacent reef fish assemblage.

Despite an eight-times greater distance, there was greater similarity between 200 and 400 m sites than between 0 and 25m sites. A substantial part of the reef influence was removed within the first 25m and, after 200m, reef proximity had very little influence on assemblage structure. Previous studies have recorded similar results. Vanderklift et al. [34] found that densities of small predatory fish 30 m from reef were significantly different to those found directly adjacent to the reef, while there was no significant difference between 30 and 300 m.

There was also considerable small-scale variability between sites that was not associated with distance from reef. In particular, the reef halo assemblage varied between sites: this variability (i.e. β diversity) has been identified as an important contributor to biodiversity in this marine park [14], [15]. One site, in particular, was different to the other three sites in the intermediate distance samples (100 m, 200 m), but at 400 m grouped closely with the other 400 m sites (Figs. 2, 3). While this site is at a similar depth, and a similar position on the shelf, it may be more exposed to currents, facilitating higher species richness at intermediate distances through bait plume dispersal and consequent attraction (Fig. 4). BRUVs studies on pelagic fish [55] and deep sea scavengers [56] have demonstrated the importance of current velocity in spreading bait plumes from BRUVs baits. Alternatively, or in conjunction, there may have been very small patches of exposed rock or boulders within the sampling area that had not been detected at the scale of the swath mapping (which had an along-track resolution of 0.75 m). Any small patches of rock<0.5 m were unlikely to be mapped as reef but, if present, may have provided habitat for some reef-associated species.

A relatively high diversity of fish species was recorded when compared with previous studies in this marine park using BRUVs (76 species from 72 replicates). Malcolm et al. [25] recorded 56 species in reef environments, although with fewer BRUVs replicates (n = 32). Species accumulation curves [25] suggested that more species would be recorded with increasing replication. Another BRUVs study in this marine park [16] recorded 137 fish species from 168 BRUVs replicates, across reef habitats from shallow (<25 m) to deep (60 m+). Other BRUVs sampling (Malcolm unpub. data) on soft sediments in the SIMP recorded a very different suite of species to reef fish assemblages previously recorded in the SIMP. Comparatively high species richness in our study likely reflects the overlap of reef and sediment species, as well as species specialising in the halo around the reef. Previous studies have demonstrated that reef fringes, or areas with mosaics of habitat, can support high diversity [57]–[60]. For example, [60] showed that mixed sand and reef recorded a higher diversity than sand or reef alone. In this study, the highest species richness was recorded at 25 metres (41 species), followed by 0 m (40 species), with overall species richness then progressively decreasing at increasing distance from reef.

### Individual Taxa

Despite the relatively high diversity recorded in this study, only a small percentage of species consistently contributed to the observed variation in assemblage structure, which is consistent with other studies [15]–[16], [25], [61]. This included: snapper *P. auratus* and mado *A. strigatus*, which were abundant at reef sites, but less so in the halo and absent from sediment habitat; and whiting *S. ciliata* and longspine flathead *P longispinis*, which were abundant in sediment and halo habitats, but uncommon or absent at sites on or adjacent to reef (0, 25 and 50 m).

Mado, *A. strigatus*, a schooling, reef-associated planktivore, was the most abundant species at 0 m but was highly variable between sites (Fig. 4), which is consistent with other studies in this marine park [17], [25]. Snapper, *P. auratus*, was mostly associated with reef, with highest abundance at 25 and 50 m, but also contributed to the halo assemblage at 100 and 200 m (Fig. 4). Snapper have been shown to be both vagile and resident in behaviour [62], [63], with home ranges measured in 100s of metres [62], [63], and this foraging away from reef was therefore expected. The sediment-associated species, whiting *S. ciliata* and longspine flathead *P. longispinis*, were absent from reef sites, and were most abundant in the halo assemblage and at 400m (Fig. 4). It is possible that competitive interactions with foraging reef fish prevented these species from approaching the reef edge, although this clearly remains speculative.

There was an inverse correlation between snapper *P. auratus* and whiting *S. ciliata* (Pearson, *r* = −0.415, P<0.001), which were two of the species most influential in discriminating between sedimentary and reef assemblages. However, this varied in relation to distance from reef. *S. ciliata* was recorded at 200 m at three sites, and at 100 m at two sites, but only in the absence of *P. auratus*. The reasons for this remain unclear but could include: the unknown presence of very small patches of reef (as previously discussed) and consequent interaction with reef fish assemblages; the aggressive nature and feeding behaviour of *P. auratus*, particularly when in higher densities.

There are no previous studies on the effects of reef proximity on abundance of flatheads (Platycephalidae). Of the six species recorded in this study (*Platycephalus arenarius, P. caeruleopunctatus, P. fuscus, P. longispinis, P. richardsonii, Ratabulus diversidens*), none were recorded on reef sites, showing a steady increase in abundance with increasing distance from reef. We considered the possibility for platycephalids to concentrate predation effort closer to reef in pursuit of small reef-associated fish, but our theory was not supported by this study. Species from this family were highly responsive to BRUVs, and this method is likely to be suitable for further specific investigations of platycephalids in these habitats.

### BRUVS as a Sampling Tool for Unconsolidated Habitats

There have been few studies using BRUVs in unconsolidated shelf habitats, and most research on fish assemblages in Australian waters in these habitats has been through netting [5], [64]–[65], or trawling [20]–[21]). However, the extractive nature of trapping and trawling is often undesirable in areas such as marine parks, and recent studies suggest that BRUVs may be a suitable alternative method of generating data in these habitats. A study in inter-reefal waters of the Great Barrier Reef Marine Park [26] found that BRUVs and prawn (shrimp) trawls sampled significantly different components of the fish assemblage, but both techniques were effective at discriminating between site groups. Their BRUVs recorded larger, more mobile, species that were able to evade trawls, such as elasmobranchs and larger teleost species of high conservation value. Eight species of chondrichthyans were recorded in this study, and BRUVs may thus be a useful method for assessing these taxa in sedimentary habitats more widely in this region.

Within the SIMP, there are vast areas of unconsolidated sediments which, as they are below the depth of access by SCUBA, are poorly described in terms of biotic assemblages and ecology [46], [48], [66]. BRUVs are a cost-effective method [67] which would appear to be ideal for rapidly gathering information on fish assemblages in these habitats. Our study has demonstrated that, when used in conjunction with high-resolution bathymetric images and GIS software for site selection, BRUVs are a powerful method for testing hypotheses related to differences in fish assemblage structure over a range of scales (10s of metres to kilometres).

### Conclusions

The influence of rocky reef on sedimentary fish assemblages in this region was strongest within 25 m of reef, diminishing rapidly thereafter. Between 100 and 200 m, there was an apparent halo effect, dominated by sedimentary fishes but still with some reef-associated species. By 400 m there was no distinguishable reef influence. Broader-scale studies targeting fish assemblages of unconsolidated habitats should, therefore, maintain a minimum distance of 400 m from any reef structures to minimise influence of reef species, unless the intention is to include the halo assemblage.

This study extends current knowledge of demersal fish assemblages within this marine park, and provides important data for the design of further studies on fish assemblages of sedimentary habitats. High-resolution bathymetric and backscatter maps were effective aids to the design of the study, especially at the smallest spatial scales. In combination, these methods enable broader examination of sedimentary fishes at various spatial and temporal scales, including cross-shelf and depth-associated patterns, and the assessment of fidelity with different types of sediment. These data will facilitate the informed conservation management of this dominant habitat both regionally and more broadly.
